# Intergenerational continuity of cell shape dynamics in *Caulobacter crescentus*

**DOI:** 10.1038/srep09155

**Published:** 2015-03-17

**Authors:** Charles S. Wright, Shiladitya Banerjee, Srividya Iyer-Biswas, Sean Crosson, Aaron R. Dinner, Norbert F. Scherer

**Affiliations:** 1 James Franck Institute, The University of Chicago, Chicago IL 60637; 2 Institute for Biophysical Dynamics, The University of Chicago, Chicago IL 60637; 3 Department of Biochemistry and Molecular Biology, The University of Chicago, Chicago IL 60637; 4 Department of Chemistry, The University of Chicago, Chicago IL 60637

## Abstract

We investigate the intergenerational shape dynamics of single *Caulobacter crescentus* cells using a novel combination of imaging techniques and theoretical modeling. We determine the dynamics of cell pole-to-pole lengths, cross-sectional widths, and medial curvatures from high accuracy measurements of cell contours. Moreover, these shape parameters are determined for over 250 cells across approximately 10000 total generations, which affords high statistical precision. Our data and model show that constriction is initiated early in the cell cycle and that its dynamics are controlled by the time scale of exponential longitudinal growth. Based on our extensive and detailed growth and contour data, we develop a minimal mechanical model that quantitatively accounts for the cell shape dynamics and suggests that the asymmetric location of the division plane reflects the distinct mechanical properties of the stalked and swarmer poles. Furthermore, we find that the asymmetry in the division plane location is inherited from the previous generation. We interpret these results in terms of the current molecular understanding of shape, growth, and division of *C. crescentus*.

Cell shape both reflects^1^ and regulates^2^ biological function. The importance of cell shape is exemplified by bacteria, which rely on specific localization of structural proteins for spatiotemporal organization^3^. Bacteria take forms resembling spheres, spirals, rods, and crescents. These shapes are defined by cell walls^4^ consisting of networks of glycan strands cross-linked by peptide chains to form a thin peptidoglycan meshwork^5^. Super-resolution imaging is now revealing the internal positions of associated proteins^6^. These include cytoskeletal proteins such as MreB, a homolog of actin^7^,^8^,^9^,^10^, intermediate filament-like bundles of CreS (crescentin)^11^,^12^, and FtsZ, a homolog of tubulin^13^. However, due to the inherently stochastic nature of molecular processes, understanding how these proteins act collectively to exert mechanical stresses and modulate the effects of turgor pressure and other environmental factors requires complementary methods such as high-throughput, quantitative optical imaging.

Multigenerational imaging data for bacterial cells can now be obtained from microfluidic devices of various designs^14^,^15^,^16^,^17^,^18^. Still, a common limitation of most devices is that the environmental conditions change throughout the course of the experiment, particularly as geometric growth of the population results in crowding of the experimental imaging spaces. We previously addressed this issue by engineering a *C. crescentus* strain in which cell adhesion is switched on and off by a small molecule (and inducible promoter)^19^, allowing measurements to be made in a simple microfluidic device^19^,^20^,^21^,^22^. This technology allows imaging >100 generations of growth of an identical set of 250–500 single cells distributed over ~25 fields of view. Thus cell density is low and remains constant. These studies afforded sufficient statistical precision to show that single *C. crescentus* cells grow exponentially in size and divide upon reaching a critical multiple (≈1.8) of their initial sizes^19^. Satisfaction of a series of scaling laws predicted by a simple stochastic model for exponential growth indicates that these dynamics can be characterized by a single time scale^19^,^23^.

In this paper, we use more advanced image analysis methods to extract cell shape contours from these data. The resulting geometric parameters, together with mathematical models, provide insights into growth and division in *C. crescentus* and the plausible role of cell wall mechanics and dynamics in these processes. Specifically, we identify natural variables for tracking cell dynamics, and develop a minimal mechanical model that shows how longitudinal growth can arise from an isotropic pressure. We then examine the dynamics of cell constriction and unexpectedly find that it is governed by the same time constant as exponential growth. This important finding can be understood in terms of an intuitive geometric model that relates the constriction dynamics to the kinetics of the growth of septal cell wall. We further suggest that the site of constriction can arise from differences in materials properties of the poles and show that it is established in the previous generation—i.e., the location of the site of division can be predicted before formation of the divisome. We relate our results to the known dynamics of contributing molecular factors and existing models for bacterial growth and division.

## Results

### The length is sufficient to characterize the exponential growth of each cell

**Figure 1:**
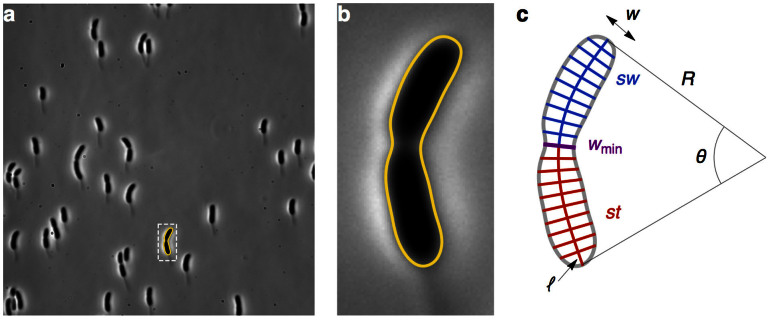
Determination of cell contour and definition of shape parameters. (a) A representative phase contrast image of one field of view. The solution flow in the microfluidic channel is from bottom to top. (b) Zoomed image of the yellow highlighted cell from a and its splined contour. (c) Schematic of a contour illustrating the shape parameters. The cell medial axis is calculated from pole to pole; it defines both cell length *ℓ*(*ϕ*) and radius of curvature *R*(*ϕ*), which lead directly to the spanning angle *θ*(*ϕ*). The cell width *w*(*ϕ*, *u*) is a parametric quantity, calculated as the length of the rib perpendicular to the medial axis at a specified distance from the stalked pole, *u*(*ϕ*). The location of the global minimum of the width *w*_min_(*ϕ*) (purple line) can be used to segment the cell into stalked (*st*, red) and swarmer (*sw*, blue) portions.

Various techniques have been put forth to analyze cell morphology gathered from single cell images^24^. Recent work on image analysis of single cells has attempted to optimize two problems: separation of distinct (but potentially overlapping) cells and accurate determination of the edge of each cell^25^. Because crowding is not an issue in our setup, we could focus solely on constructing an algorithm to delineate each cell contour accurately and precisely. As shown in Fig. 1a,b and described in the Methods section, we first segment each cell using pixel-based edge detection similar to Ref. 26, then perform spline interpolation to determine the cell contour at sub-pixel resolution. The sequence of such images for each single cell constitutes a trajectory in time *t* that serves as the basis for quantitative analysis. Division events are then detected in an automated fashion using custom Python code, and used to divide time trajectories for each cell into individual generations.

All data shown here were obtained by observing 260 single *C. crescentus* cells perfused in complex medium (peptone-yeast extract; PYE) at 31°C over the course of 2 days (corresponding to 9672 separate generations). Under these conditions, the mean population growth rate and division time remain constant, so we treat the trajectories of individual generations as members of a single ensemble. In other words, we segment each cell trajectory by generation and take the resulting initial frame (i.e., immediately following division) as *t* = 0 minutes. In order to average over the ensemble, we then bin quantitative information according to time since division, *t*, normalized by the respective division time *τ*. The normalized time, *ϕ* ≡ *t*/*τ*, serves as a cell-cycle phase variable.

**Figure 2:**
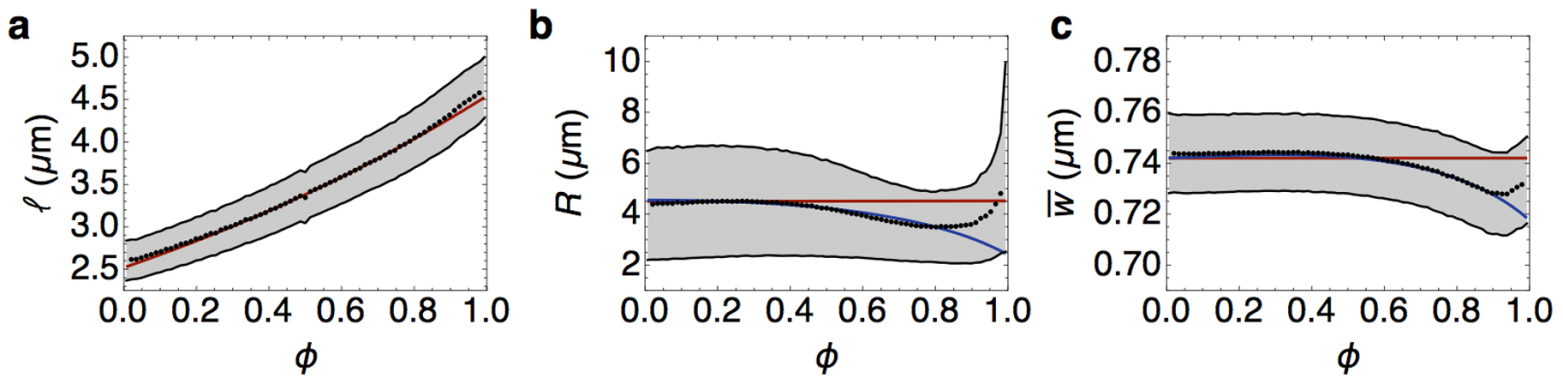
Dynamics of cell shape parameters. (a) Length of the cell medial axis (data shown in black and exponential fit from our mechanical model in red). (b) Radius of curvature of the cell medial axis, obtained by calculating the best-fit circle to the entire cell (black points), with a time-averaged mean 〈*R*〉 = 4.44 ± 2.12 *μ*m (0 < *ϕ* < 0.5). The mean-field model predicts a constant steady-state value for 〈*R*(*ϕ*)〉 (red solid line), whereas by accounting for constriction dynamics, the model captures the dip in 〈*R*(*ϕ*)〉 seen for 0.5 < *ϕ* < 0.9 (blue solid line). (c) Characteristic cell width, obtained by spatially averaging the width at each time point (black points), with a time-averaged mean 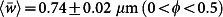. The mean field model predicts a constant steady-state value for 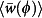 (red solid line), whereas cell constriction accounts for the dip in 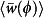 seen for *ϕ* > 0.5 (blue solid line). The shaded regions represent ±1 standard deviation. Model parameters: *P* = 0.3 MPa, *γ* = 50 nN/*μ*m, *k_c_* = 2 nN*μ*m^2^, *R_c_* = 0.5 *μ*m, *k_m_* = 40 nN*μ*m, *R_m_* = 0.31 *μ*m, *τ* = 73 min.

For our quantitative analysis, we focus on a set of three intuitive and independent parameters that characterize cell shape at each stage of growth: length *ℓ*, width *w*, and radius of curvature *R* (Fig. 1c). They are calculated directly from each splined contour as follows (see also Supplementary Fig. 1):

- We define the length, *ℓ*(*ϕ*), as the pole-to-pole distance along the contour of the cell medial axis at the normalized time *ϕ* (Fig. 2a).
- We assign a single radius of curvature, *R*(*ϕ*), to each cell based upon the best-fit circle to the medial axis (Fig. 2b). Although stalked (*R^st^*(*ϕ*)) and swarmer (*R^sw^*(*ϕ*)) portions may be described by different radii of curvature toward the end of the cell cycle, the average radius obtained by averaging the contributions of each portion yields the same value, i.e., 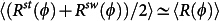 (see Supplementary Fig. 2c).
- We define the width, *w*(*ϕ*, *u*) as the length of the perpendicular segment spanning from one side of the cell contour to the other at each position *u*(*ϕ*) along the medial axis, which runs from *u* = 0 at the stalked pole to *u* = *ℓ* at the swarmer pole. Furthermore, we spatially averaged the width over positions along the medial axis, 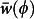, to obtain a characteristic width at each time point (Fig. 2c).

The mean division time is 〈*τ*〉 = 73 ± 7 min, where 〈…〉 indicates a population average. We find that 〈*ℓ*(*ϕ*)〉 increases exponentially with time constant 〈*κ*〉^−1^ = 125 ± 8 min, essentially the same time constant that we previously observed for the cross-sectional area^19^, while 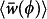 and 〈*R*(*ϕ*)〉 remain approximately constant for 0 < *ϕ* < 0.5 and each shows a dip for 0.5 < *ϕ* < 0.9 when cell constriction becomes prominent. The sharp rise in 〈*R*(*ϕ*)〉 seen for *ϕ* > 0.9 results from independent alignment of the stalked and swarmer portions with the microfluidic flow as they become able to move independently (i.e., fluctuate easily about the plane of constriction). These observations confirm the assumptions in Ref. 19 that the length is sufficient to describe the growth of cell size. Moreover, we can track the dynamics of the spanning angle, *θ*, using the relation *ℓ* = *Rθ*.

### Mechanical model for cell shape and growth

There are many details of cell growth and shape that require interpretation. For example, it is not obvious *a priori* that growth should be almost exclusively longitudinal. Therefore, we have developed a minimal mechanical model that can explain these observations. We parametrize the geometry of the cell wall by a collection of shape variables {*q_i_*(*t*)}, where *q*_1_ = 〈*R*〉, 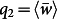, and *q*_3_ = 〈*θ*〉 are the parameters introduced above (Fig. 1c). As the cell grows in overall size, we postulate that the rate of growth in the shape parameter *q_i_*(*t*) is proportional to the net decrease in cell wall energy, *E*({*q_i_*(*t*)}), per unit change in *q_i_*(*t*)^27^,^28^. Assuming linear response, the configurational rate of strain, 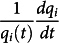, is proportional to the corresponding driving force *F_i_* = −*∂E*/*∂q_i_*, in analogy with the constitutive law of Newtonian flow^29^:
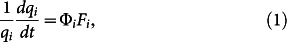
where the constant Φ*_i_* describes the rate of irreversible flow corresponding to the variable *q_i_*(*t*). According to equation (1), exponential growth occurs if *F_i_* is constant, whereas *q_i_*(*t*) reaches a steady-state value if *F_i_*(*q_i_*) = 0 along with the condition *∂F_i_*/*∂q_i_* < 0. It thus remains to specify the form of *E*.

For a *C. crescentus* cell of total volume *V* and surface area *A*, our model for the total energy in the cell wall is given by
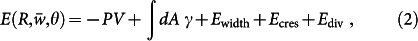
where *P* is a constant pressure driving cell wall expansion; *γ* is the tension on the surface of the cell wall; *E*_width_ is the energy required to maintain the cell width; *E*_cres_ represents the mechanical energy required to maintain the crescent cell shape; *E*_div_ is the energy driving cell wall constriction. Traditionally *P* was taken to be the turgor pressure^27^; while the importance of the turgor pressure has recently been questioned^30^, an effective pressure must still arise from the synthesis and insertion of peptidoglycan strands that constitute the cell wall. We note that a purely elastic description of cell wall mechanics would lead to a curvature-dependent surface tension^31^. However, if growth is similar to plastic deformation, the tension is uniform^32^. The effective tension in our model depends on the local surface curvatures through the energy terms *E*_width_ and *E*_cres_, that describe harmonic wells around preferred values of surface curvatures.

The mechanical energy for maintaining width is given by
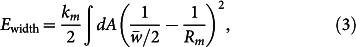
where the constant *R_m_* is the preferred radius of curvature, *k_m_* is the bending rigidity and *dA* is a differential area element^33^. Contributions to *k_m_* can come from the peptidoglycan cell wall as well as membrane-associated cytoskeletal proteins like MreB, MreC, RodZ, etc., which are known to control cell width^7^,^8^,^9^.

In addition to maintaining a constant average width, *C. crescentus* cells exhibit a characteristic crescent shape, which relies on expression of the intermediate filament-like protein crescentin^11^. Although the mechanism by which crescentin acts is not known, various models have been proposed, including modulation of elongation rates across the cell wall^12^,^34^ and bundling with a preferred curvature^35^. We assume the latter and write the energy for maintaining the crescent shape as
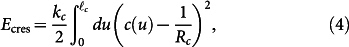
where *u* is the arc-length parameter along the crescentin bundle attached to the cell wall, *c*(*u*) is the local curvature, *R_c_* is the preferred radius of curvature, *ℓ_c_* is the contour length, and *k_c_* is the linear bending rigidity. Equation (4) accounts for the compressive stresses generated by the crescentin bundle on one side of the cell wall, leading to a reduced rate of cell growth, according to equation (1). As a result, the cell wall grows differentially and maintains a non-zero curvature of the centerline. In the absence of crescentin (*k_c_* = 0), our model predicts an exponential decay in the cell curvature that leads to a straight morphology, consistent with previous observations.^11^

Finally, one must also account for the energy driving cell wall constriction. Constriction proceeds via insertion of new peptidoglycan material at the constriction site. This process leads to the formation of daughter pole caps^36^. We take constriction to be governed by an energy of the form *E*_div_ = −*λS*, where *S* is the surface area of the septal cell wall, and *λ* is the energy per unit area released during peptidoglycan insertion.

### There exists an optimal cell geometry for a given mechanical energy

To apply the model introduced above (equations (1) and (2)) to interpreting the data in Fig. 2, we assume a minimal cell geometry given by a toroidal segment with uniform radius of curvature *R*(*ϕ*), uniform cross-sectional width 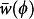 and the spanning angle *θ*(*ϕ*). To this end, we estimate as many mechanical parameters as we can from the literature and then determine the rest by fitting our experimentally measured values. Turgor pressure in Gram-negative bacteria has been measured to be in the range 0.03–0.5 MPa^37^,^38^,^39^. We use a value for the effective internal pressure close to the higher end of the measured values for turgor pressure, *P* = 0.3 MPa, in order to account for peptidoglycan insertion. We estimate the surface tension as *γ* = 50 nN/*μ*m (see Supplementary model section) and multiply it by the cell surface area 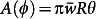 to obtain the cell wall surface energy. First, we neglect cell constriction (setting *E*_div_ = 0) and assume that the crescentin structure spans the length of the cell wall (excluding the endcaps)^40^, with a contour length 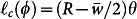. The mechanical properties of MreB and crescentin are likely similar to those of F-actin and intermediate filaments, respectively^11^,^41^. However, due to a lack of direct measurements, we obtain the mechanical parameters *k_m_* and *k_c_* by fitting the model to the experimental data.

As desired, we find that the total energy *E* has a stable absolute minimum at particular values of the cross-section diameter 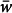 and the centerline radius *R*, given by solution of 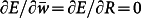 (see Supplementary Fig. 4). The measured values are 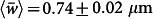 and 〈*R*〉 = 4.44 ± 2.12 *μ*m (0 < *ϕ* < 0.5), and, as indicated by the red solid curves in Fig. 2b,c, the model reproduces them with *k_m_* = 40 nN*μ*m and *k_c_* = 2 nN*μ*m^2^. While the fitted value for *k_c_* is numerically close to the estimate based on the known mechanical properties of intermediate filaments (~1.5 nN*μ*m^2^), the value for *k_m_* is much higher than the bending rigidity of MreB bundles (see Supplementary Information). This indicates that *k_m_* is only determined in part by MreB and can have contributions from the cell wall.

Given stable values for 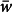 and *R*, growth is completely described by the dynamics of the angle variable *θ*(*ϕ*). Consequently, we write the total energy in the scaling form 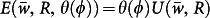, with *U* the energy density along the longitudinal direction. The condition for growth then becomes *U* < 0, such that the energy is minimized for increasing values of *θ*(*ϕ*). From our experimental data, the angle spanned by the cell centerline increases by an amount 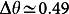 during the entire cycle. Using our parameter estimates and fitting the data in Fig. 2, we obtain a numerical value for the energy density 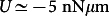. We relate the angle dynamics to the length by
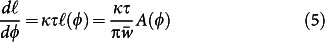
where *κ* = −Φ*_θ_U* (*U* < 0) is the rate of longitudinal growth, which can be interpreted as resulting from remodeling of peptidoglycan subunits with a mean current 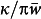, across the cell surface area *A*(*ϕ*). From an exponential fit to the data for cell length (Fig. 2a), we obtain Φ*_θ_* = 1.6 × 10^−3^ (nN *μ*m min)^−1^, which gives us an estimate of the friction coefficient, 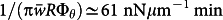, associated with longitudinal growth; e.g., MreB motion that is known to correlate strongly with the insertion of peptidoglycan strands.^42^ Our results are consistent with previous observations of *C. crescentus* cells with arrested division but continued growth^43^.

### Constriction begins early and proceeds with the same time constant as exponential growth

Having characterized the dynamics of growth, we now turn to constriction at the division plane. As mentioned above, we obtain the experimental width at each point along each cell's medial axis. The typical width profile is non-uniform along its length, exhibiting a pronounced invagination near the cell center (with width *w*_min_(*ϕ*); Fig. 1c). This invagination, which ultimately becomes the division plane, is readily identifiable early in the cell cycle, even before noticeable constriction occurs. We discuss the kinetics of constriction in this section, and focus on its location later in the manuscript. As shown in Fig. 3a (black points), 〈*w*_min_(*ϕ*)〉 progressively decreases towards zero until pinching off at *ϕ* = 1. Due to the limited spatial resolution of our imaging (phase contrast microscopy), the pinch-off process occurring for *ϕ* > 0.9 could not be captured, but 〈*w*_min_(*ϕ*)〉 at earlier times (i.e., *ϕ* < 0.9) is precisely determined as a function of *ϕ*.

To model the dynamics of constriction, we assume as in Ref. 44 (Fig. 3a, inset): (i) the shape of the zone of constriction is given by two intersecting and partially formed hemispheres with radii *w*_max_/2; and (ii) constriction proceeds by completing the missing parts of the hemisphere such that the newly formed cell wall surface maintains the curvature of the pre-formed spherical segments. As a result, a simple geometric formula is obtained that relates the width of the constriction zone, *w*_min_(*ϕ*), to the surface area *S*(*ϕ*) of the newly formed cell wall,
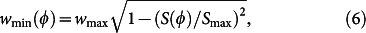
where 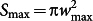 is the maximum surface area achieved by the caps as the constriction process is completed, i.e., when *w*_min_(*ϕ* = 1) = 0. We assume that the addition of new cell wall near the division plane initiates with a rate, *κ*_0_, and thereafter grows exponentially with a rate, *κ_d_*, according to,
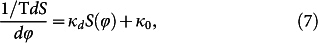
subject to the initial condition *S*(*ϕ* = 0) = 0. The first term on the right-hand side of equation (7) follows from equation (1), using *S*(*ϕ*) as the shape variable, after incorporating the constriction energy *E*_div_(*ϕ*). The rate of septal peptidoglycan synthesis, *κ_d_*, is thus directly proportional to the energy per unit area released during constriction, *λ*. The solution, 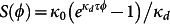, can then be substituted into equation (6) to derive the time-dependence of *w*_min_(*ϕ*), whose dynamics is controlled by two time scales: 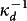 and 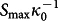. Fitting equation (6) with the data for 〈*w*_min_(*ϕ*)〉, we obtain 
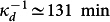
 and 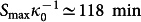. The fitted values for the time constants controlling constriction dynamics (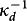 and 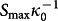) are remarkably similar to that of exponential cell elongation (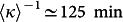). This shows that septal growth proceeds at a rate comparable to longitudinal growth. Therefore, one of the main conclusions that we draw is that cell wall constriction (Fig. 3a) is controlled by the same time constant as exponential longitudinal growth (Fig. 2a).

Having determined the dynamics of *w*_min_(*ϕ*), we compute the average width across the entire cell 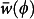 using the simplified shape of the constriction zone as shown in Fig. 3a (inset). The resultant prediction (blue solid curve in Fig. 2c) is in excellent agreement with the experimental data and captures the dip in 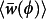 seen for *ϕ* > 0.5. Constriction also leads to a drop in the average radius of curvature of the centerline, as shown by the experimental data in Fig. 2b. In the supplementary material we derive a relation between the centerline radius of curvature *R*(*ϕ*) and the minimum width *w*_min_(*ϕ*), given by 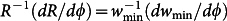, predicting that cell curvature increases at the same rate as *w*_min_(*ϕ*) drops. Using this relation, we are able to quantitatively capture the dip in 〈*R*(*ϕ*)〉 seen for *ϕ* > 0.5 (solid blue curve in Fig. 2b) without invoking any additional fitting parameters.

### Origin of the asymmetric location of the primary invagination

We now consider the position of the division plane and its interplay with cell shape. As shown in Fig. 3b, the distance of the width minimum from the stalked pole (*ℓ^st^*(*ϕ*)) increases through the cell cycle at the same rate as the full length of the growing cell (*ℓ*(*ϕ*)), such that their ratio remains constant with time-averaged mean 〈*ℓ^st^*/*ℓ*〉 = 0.54 ± 0.05. The presence of the primary invagination early in the cell cycle is reiterated in Fig. 3c, which shows the width profile constructed by ensemble-averaging over each cell at the timepoint immediately following division. In addition to the width minimum *w*_min_(*ϕ*), there are two characteristic maxima near either pole, 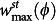 and 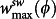, respectively (Fig. 3c, inset). As evident in Fig. 3c, the stalked pole diameter 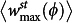 is on average larger than its swarmer counterpart 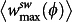 (also see Supplementary Fig. 2a).

We show that the asymmetric location of the invagination (and the asymmetric width profile) can originate from the distinct mechanical properties inherent to the pole caps in *C. crescentus*. The shapes of the cell poles can be explained by Laplace's law that relates the pressure difference, *P*, across the cell wall to the surface tensions in the stalked or the swarmer pole, 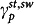. The radii of curvature of the poles then follow from Laplace's law
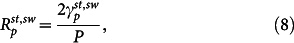
where the superscript (*st*, *sw*) denotes the stalked or the swarmer pole. Thus a larger radius of curvature in the poles has to be compensated by a higher surface tension to maintain a constant pressure difference *P*. Assuming that the poles form hemispheres, we have 
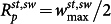
. Our data indicate that the early time ratio for 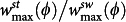 (*ϕ* < 0.1) shows a strong positive correlation with the ratio *ℓ^st^*(*ϕ*)/*ℓ^sw^*(*ϕ*), with an average value 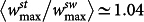 (see Supplementary Fig 3a). Laplace's law then requires that the stalked pole be mechanically stiffer than the swarmer pole; 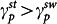. This observation suggests that the asymmetry in the lengths of the stalked and swarmer parts of the cell depends upon different mechanical properties of the respective poles.

To quantitatively support this claim, we investigate an effective contour model for the cell shape. To this end, we assume that the fluctuations in cell shape relax more rapidly than the time scale of growth. This separation of timescales allows us to derive the equation governing the cell contour by minimizing the total mechanical energy (equation (2)). From the solution we compute the resultant width profile for the entire cell (see Supplementary model section). As shown in Fig. 3d, the model with asymmetric surface tensions of the poles causes the primary invagination to occur away from the cell mid-plane. The spatial location of the invagination relative to the cell length depends linearly on the ratio 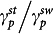. Symmetry is restored for 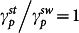, as shown in Fig. 3d (blue dashed curve). We note that a gradient in *γ* along the cell body would imply differences in longitudinal growth rates between the stalked and the swarmer portions of the cell (Eq. (1)). Our data exclude this possibility since both *ℓ^st^*(*ϕ*) and *ℓ^sw^*(*ϕ*) grow at the same rate *κ*, as evidenced by the constancy of their ratio (Fig. 3b and Supplementary Fig. 2d). Because *C. crescentus* does not exhibit polar growth, the *polar stiffness model* is consistent with the observed uniformity in longitudinal growth rate. In addition, the non-uniformity in cell width comes from the differences in mechanical response in the cell wall due to preferential attachment of crescentin along the concave sidewall. For a creS mutant cell (where *k_c_* = 0), our model predicts a uniform width profile before the onset of constriction.

### Cell shape evolution during wall constriction

**Figure 4:**
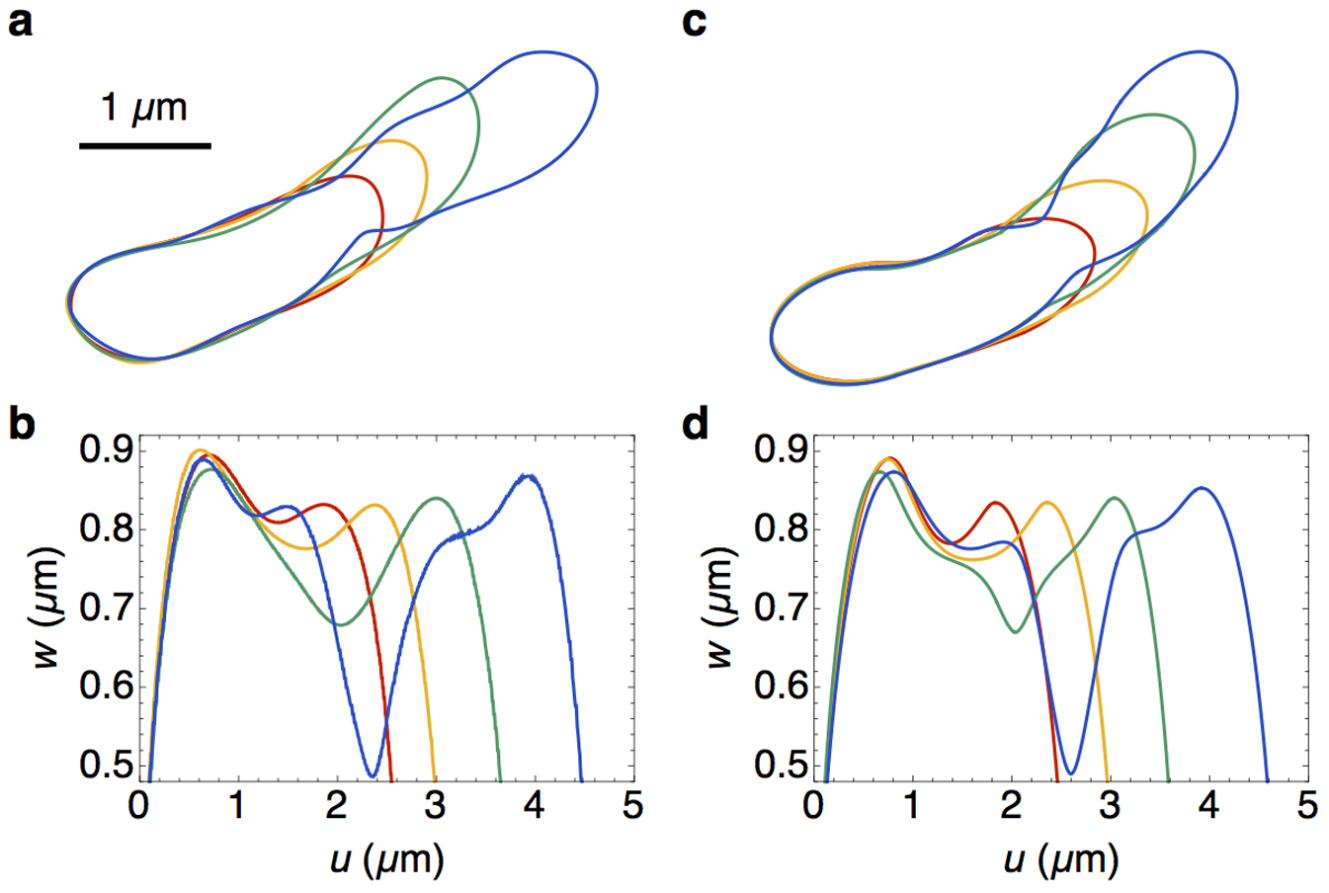
Comparison of experimental and model cell contours and width profiles. (a) Splined contours of a growing and constricting cell at different values of normalized time *ϕ* = 0.0 (red), 0.33 (orange), 0.67 (green) and 1.0 (blue). (b) Experimental width profiles plotted against absolute distance from the stalked pole, corresponding to contours in a. (c) Contours computed from the cell shape model at different values of *w*_min_ and *ℓ* corresponding to the time points in a. (d) Model width profiles corresponding to the contours in c.

**Figure 5:**
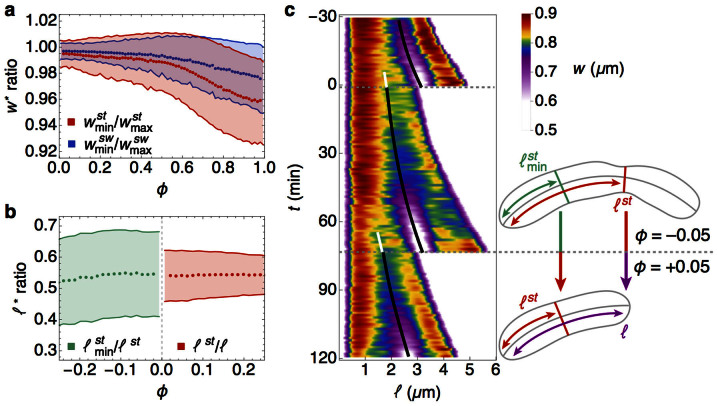
Location of division plane is set in the previous generation. (a) Value of the minimum width normalized by the respective maximum width for the stalked (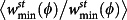) and the swarmer (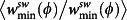) parts. (b) Ratio of length from stalked pole to secondary minimum normalized by length from stalked pole to primary minimum 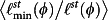, green) and ratio of length from stalked pole to primary minimum normalized by total length (〈*ℓ^st^*(*ϕ*)/*ℓ*(*ϕ*)〉, red). The former ratio remains constant at 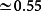, while the latter obtains this value at the end of the cell cycle. In comparing averages between two generations here, we indicate values of *ϕ* from the first generation as negative (i.e., *ϕ* = −0.2 is 20% of the way from the subsequent division). The green points are not shown for *ϕ* < −0.25 due to increased errors in identification. (c) Kymograph of width profiles for a typical cell over two generations. The time evolution of the widths (color scale) illustrates continuity of the location of the minima across generations. That is, the location of the secondary minimum just before division (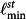, white line) becomes the primary minimum (*ℓ^st^*, black line) just after division (horizontal dashed line). The schematic at right shows two measured contours that correspond to time points immediately before (*ϕ* = −0.05) and after (*ϕ* = +0.05) the division event shown in the kymograph.

The experimental width profiles show that the growing and constricting cells typically develop a second minimum in width (Fig. 4a,b). These secondary invaginations are observed in both the stalked and swarmer portions of single cells in the predivisional stage (*ϕ* > 0.6), although they are more common in the stalked portions (Fig. 5a). We show here that these secondary minima become the primary minima in each of the daughter cells. To study the dynamics of the development of the secondary minimum we introduce a new quantity, 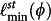, defined as the distance from the stalked pole to the secondary minimum in the stalked part (see Fig. 5c, inset). We find that the ratio 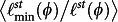 has a mean value of 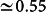 at later points in the cell cycle (Fig. 5b), equal to the constant ratio maintained by the distance from the stalked pole to the primary minimum, 〈*ℓ^st^*(*ϕ*)/*ℓ*(*ϕ*)〉. In fact the kymograph of width profiles (shown over 2 generations for a representative single cell) in Fig. 5c demonstrates that the predivisional secondary invaginations are inherited as primary invaginations after division. This mechanism provides continuity and inheritance of the invaginations across generations and is an intrinsic element of the mechanism for cell division in *C. crescentus*.

To quantitatively explain the experimental width profiles during constriction, we use our mechanical model to determine the instantaneous cell shape by minimizing the total energy (equation (2)) at the specified time points (see Supplementary model section). To take constriction into account, we impose the constraint that *w*(*ℓ^st^*, *ϕ*) = *w*_min_(*ϕ*), where *w*_min_(*ϕ*) is determined by equations (6) and (7). In addition, we assume non-uniform materials properties in the cell wall by taking the tension in the cell poles (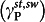) and the septal region to be higher than the rest of the cell. As constriction proceeds and *w*_min_(*ϕ*) decreases, we compute the shape of the cell contours (Fig. 4c) and the corresponding width profiles (Fig. 4d). The computed width profiles faithfully reproduce the secondary invaginations, which become more pronounced as the daughter pole caps become prominent. An example of the experimental width profiles is shown in Fig. 4b at evenly-spaced intervals in time for a single generation, and the corresponding model width profiles are shown in Fig. 4d.

We note that the experimental cell contours in the predivisional stage (*ϕ* > 0.9) bend away from the initial midline axis and develop an alternate growth direction (Fig. 4a, blue contour). These bend deformations are induced by the microfluidic flow about the pinch-off plane; the cells become increasingly “floppy” as the constriction proceeds.

## Discussion and Conclusions

The consistent propagation of a specific shape through the processes of growth and division relies upon an intricate interplay between the controlled spatiotemporal expression and localization of proteins, and cytoskeletal structural elements. The high statistical precision of our measurements allows us to gain new insights into cell morphology. From precise determination of cell contours over time, we observe that a typical cell width profile is non-uniform at all times with a pronounced primary invagination appearing during the earliest stages of the cell cycle. During cell constriction, the decrease in the minimum width is governed by the same time constant as exponential axial growth (Fig. 3a). Furthermore, the location of the primary invagination divides the cell contour into its stalked and swarmer compartments, such that the ratio of the length of the stalked part *ℓ^st^*(*ϕ*) to the total pole-to-pole length *ℓ*(*ϕ*) remains constant during the cycle with a mean value 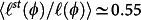 (Fig. 3b). These observations and our mechanical model lead to two important conclusions: first, *the dynamics of cell wall constriction and septal growth occur concomitantly*, and second, *the asymmetric location of the primary invagination can be explained by the differences in mechanical properties in the stalked and swarmer poles*. A corollary of the first conclusion is that the size ratio threshold at division occurs naturally without requiring a complex timing mechanism^19^.

In addition to the primary septal invagination, the cell contours exhibit a pronounced secondary invagination during the predivisional stages (Fig. 4). Remarkably, the secondary invaginations develop at a precise location relative to the total length of the stalked compartments, 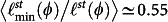 (Fig. 5b). The data thus allow a third conclusion: *these secondary invaginations are inherited as primary invaginations in each of the daughter cells, directing the formation of the division plane in the next generation*. Thus, through consistent and controlled nucleation of invaginations across generations, *C. crescentus* cells maintain a constant ratio of the sizes of stalked and swarmer daughter cells.

Our experimental observations and the parameters in the cell shape model can be related to the current molecular understanding for Gram-negative bacteria, in particular *C. crescentus*. Before the onset of noticeable constriction, cell shape is dictated by the mechanical properties of the peptidoglycan cell wall in addition to various shape-controlling proteins such as MreB, MreC, RodZ and CreS. Single molecule tracking studies have revealed that MreB forms short filamentous bundles anchored to the inner surface of the cell wall and moves circumferentially at a rate much faster than the rate of cell growth^42^,^45^. *In vitro* experiments show that MreB filaments can induce indentation of lipid membranes, suggesting that they may have a preferred radius of curvature^46^. Thus on time scales comparable to cell growth, *E*_width_ is determined in part by the energy cost of adhering MreB bundles to the cell wall (see Supplementary model section).

Bacterial cell division is driven by a large complex of proteins, commonly known as divisomes that assemble into the Z-ring structure near the longitudinal mid-plane of the cell^13^. The Z-ring contains FtsZ protofilaments that are assembled in a patchy band-like structure^47^. FtsZ protofilaments are anchored to the cell membrane via FtsA and ZipA, and play a crucial role in driving cell wall constriction^48^. During constriction, the divisome proteins also control peptidoglycan synthesis and direct the formation of new cell wall via the activity of penicillin-binding proteins (PBPs)^49^,^50^. Thus the divisome plays a two-fold role by concomitantly guiding cell wall constriction and growth of the septal peptidoglycan layer. According to our model the constriction of the cell wall is driven by the synthesis of septal cell wall at a rate *κ_d_* (~〈*κ*〉), which can be directly related to the activity of PBPs triggered by the divisome assembly. Furthermore, in our model it is sufficient that FtsZ and the divisome guide the curvature of cell wall growth in the septal region (see Fig. 3a, inset).

While the mechanism behind the precise asymmetric location of the division plane in *C. crescentus* cells is not well understood, it is likely that the ATPase MipZ helps division site placement by exhibiting an asymmetric concentration gradient during the predivisional stage^51^. MipZ activity inhibits FtsZ assembly; as a result of polar localization of MipZ, Z-ring assembly is promoted near the mid-cell^52^. Our cell shape model suggests that the early time asymmetric location of the primary invagination, which develops into the division plane, is controlled by the differences in surface tensions maintained in the poles. The presence of this invagination at *ϕ* = 0, as inherited from the secondary invaginations in the previous generation, aids in Z-ring assembly at the site of the invagination. The curvature-sensing capability of the Z-ring may be enabled by the minimization of the FtsZ polymer conformational energy that is determined by the difference between cell surface curvature and FtsZ spontaneous curvature^13^,^53^.

A higher tension in the stalked pole can be induced by asymmetric localization of polar proteins, such as PopZ, early in the cell cycle. Experiments have shown that PopZ localizes to the stalked pole during the initial phase of the cell cycle and increasingly accumulates at the swarmer pole as the cell cycle proceeds^54^. Consistent with this observation, our data show that the correlation between the pole sizes (determined by the ratio of surface tension to pressure) and the stalked and swarmer compartment lengths tend to disappear later in the cycle (Supplementary Fig. 3), as cell constriction proceeds. A recent experimental study also demonstrates that molecular perturbation of Clp proteases can destroy the asymmetry of cell division in *C. crescentus*^55^, suggesting the interplay of subcellular protease activity with the physical properties of the cell wall.

Earlier theoretical models have predicted that a small amount of pinch-off force from the Z-ring (~8 pN) is sufficient to accomplish division by establishing a direction along which new peptidoglycan strands can be inserted^27^. In contrast, our data combined with the mathematical model allows the interpretation that *the early time asymmetric invagination in the cell wall can set the direction for the insertion of new peptidoglycan strands*. Constriction results from exponential growth of surface area in the septum (at the same rate as longitudinal extension). The instantaneous cell shape is determined by minimizing the energy functional at given values of the cell size parameters.

Finally, from our estimate of the cell wall energy density 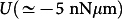, we predict that a net amount 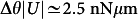 of mechanical energy is used by the peptidoglycan network for cell wall growth. For a *C. crescentus* cell of surface area 12.5–25 *μ*m^2^, layered with glycan strands of length ~5 nm and cross-linked by peptide chains with maximally stretched length ~4 nm^5^, there are roughly 10^6^ peptidoglycan subunits. Thus on average, each peptidoglycan subunit can consume mechanical energy of ~2.4 × 10^−6^ nN*μ*m, or ~0.6 *k_B_T* at a temperature *T* = 31°C. Cell wall remodeling and insertion of new peptidoglycan material can likely create defects in the peptidoglycan network^56^. One thus expects cellular materials properties to change over time, as a result of these molecular scale fluctuations. Although we neglect such variations in our mean field model, it nonetheless quantitatively captures the average trends in cell shape features. In future work we plan to more closely connect the energy terms of the continuum model with molecular details.

## Methods

### Acquisition of experimental data

Data were acquired as in Ref. 19. Briefly, the inducibly-sticky *Caulobacter crescentus* strain FC1428 was introduced into a microfluidic device and cells were incubated for one hour in the presence of the vanillate inducer. The device was placed inside a homemade acrylic microscope enclosure (39″ × 28″ × 27″) equilibrated to 31°C (temperature controller: CSC32J, Omega and heater fan: HGL419, Omega). At the start of the experiment, complex medium (peptone-yeast extract; PYE) was infused through the channel at a constant flow rate of 7 *μ*L/min (PHD2000, Harvard Apparatus), which flushed out non-adherent cells. A microscope (Nikon Ti Eclipse with the “perfect focus” system) and robotic XY stage (Prior Scientific ProScan III) under computerized control (LabView 8.6, National Instrument) were used to acquire phase-contrast images at a magnification of 250X (EMCCD: Andor iXon+ DU888 1 k × 1 k pixels, objective: Nikon Plan Fluor 100X oil objective plus 2.5X expander, lamp: Nikon C-HFGI) and a frame rate of 1 frame/min for 15 unique fields of view over 48 hours. In the present study we use a dataset consisting of 260 cells, corresponding to 9672 generations (division events).

### Analysis of single cell shape

The acquired phase-contrast images were analyzed using a novel routine we developed (written in Python). Each image was processed with a pixel-based edge detection algorithm that applied a local smoothing filter, followed by a bottom-hat operation. The boundary of each cell was identified by thresholding the filtered image. A smoothing B-spline was interpolated through the boundary pixels to construct each cell contour. Each identified cell was then tracked over time to build a full time series. We chose to include only cells that divided for more than 10 generations in the analysis. A minimal amount of filtering was applied to each growth curve to remove spurious points (e.g., resulting from cells coming together and touching, or cells twisting out of plane). The timing of every division was verified by visual inspection of the corresponding phase contrast images, so that the error in this quantity is approximately set by the image acquisition rate of 1 frame/min.

## Supplementary Material

Supplementary Information Supplementary Information

## Figures and Tables

**Figure 3:**
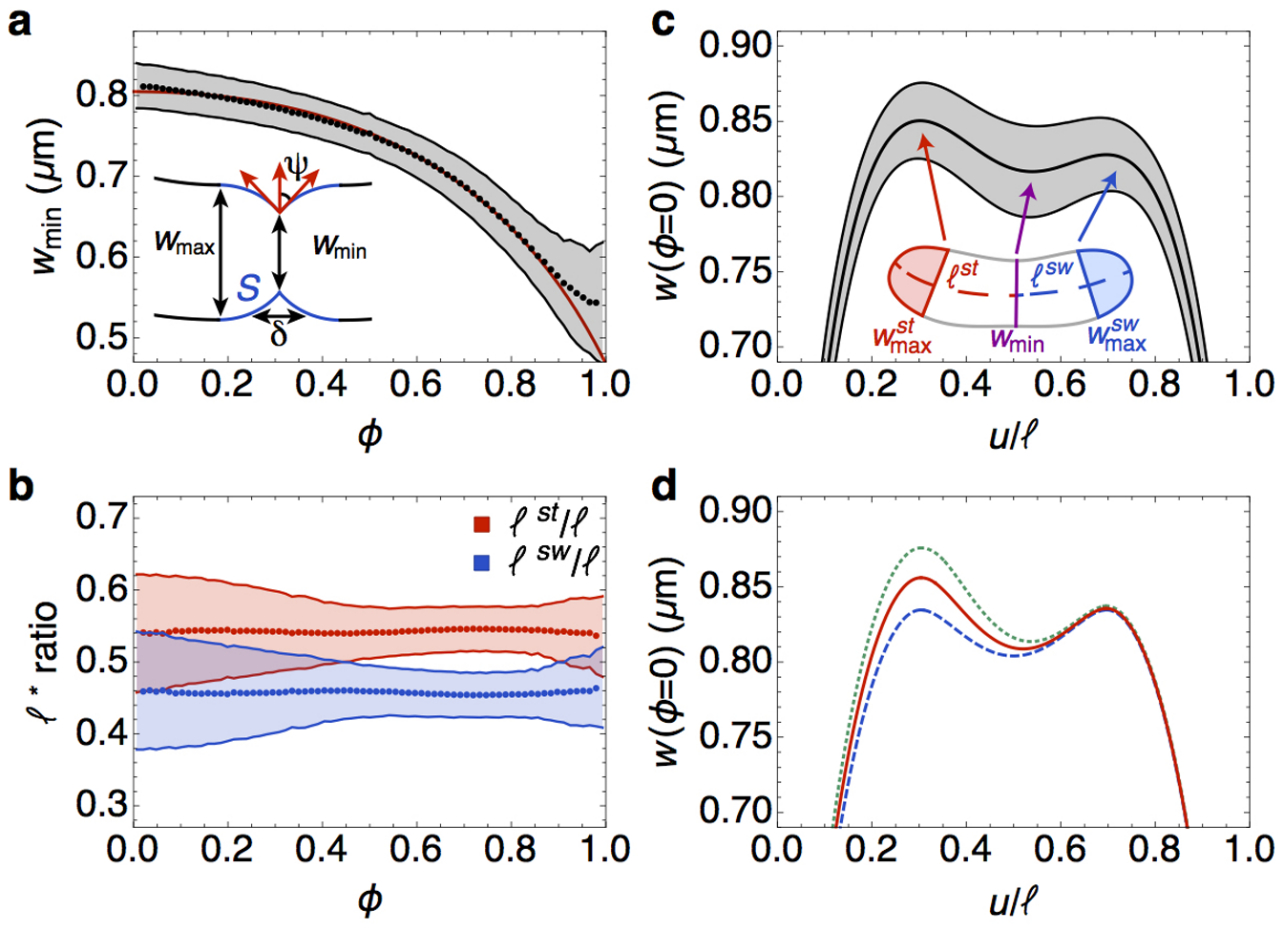
Timing and location of the division plane. (a) Time-dependence of 〈*w*_min_(*ϕ*)〉, with the experimental points in black and the model fit (equations (6) and (7)) in red. Fit values: *w*_max_ = 0.805 *μ*m, 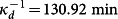, *κ*_0_ = 0.016 *μ*m^2^/min. Inset: Minimal geometry of a constricting cell, where *S* (blue) is the septal cell wall synthesized during constriction, *δ* is longitudinal width of the constriction zone, and *ϕ* is the tangent angle at constriction. (b) Ratios of the length of the stalked (red) and swarmer (blue) portions divided by the total length (〈*ℓ^st^*(*ϕ*)/*ℓ*(*ϕ*)〉 and 〈*ℓ^sw^*(*ϕ*)/*ℓ*(*ϕ*)〉, respectively), are approximately constant over the cell cycle. (c) Experimental width profile of *C. crescentus* cells in the initial stage of the cycle (*ϕ* = 0) after ensemble-averaging over all data. The width 〈*w*(*ϕ* = 0, *u*)〉 is plotted as a function of the distance from the stalked end normalized to the length of the cell, *u*/*ℓ*. Inset: A representative single cell contour immediately after division (*ϕ* = 0), showing the location of the local minimum in the width (*w*_min_, purple line) as well as two local maxima (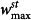, red line and 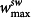, blue line). These two local maxima in the width define the pole regions (shaded in red and blue, respectively). (d) Model width profiles of the cell showing symmetric and asymmetric location of the invagination near the midplane for different values of the ratio 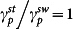 (blue, dashed), 1.05 (red, solid) and 1.09 (green, dotted). Parameter values are the same as in Fig. 2 with 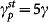
27. The shaded regions in a, b and c represent ±1 standard deviation.
