# Supplementary material for: Intergenerational continuity of cell shape dynamics in Caulobacter crescentus
Source: Sci Rep. 2015 Mar 17;5:9155. doi: 10.1038/srep09155 (PMC4894450; doi:10.1038/srep09155)
Supplement: Supplementary Information [file srep09155-s1.pdf]

Supplementary Information

Intergenerational continuity of cell shape dynamics in *Caulobacter crescentus*

Charles S. Wright, Shiladitya Banerjee, Srividya Iyer-Biswas, Sean Crosson,
Aaron R. Dinner & Norbert F. Scherer

EXPERIMENTAL CELL SHAPE PARAMETERS

A number of quantities can be immediately calculated from each splined cell contour (Supplementary Fig. 1a), including the cross-sectional area. The cell medial axis was determined by calculating the Voronoi diagram of the cell contour [1], pruning the branches (Supplementary Fig. 1b), and extending this skeleton to the edges of the cell contour in a manner that preserves average curvature of the medial axis (Supplementary Fig. 1c). The intersections between the cell medial axis and contour represent the stalked and swarmer poles, respectively. The cell length was calculated by evaluating the distance along the medial axis between either pole. The cell widths were determined by creating ribs perpendicular to the medial axis along its length and determining the distances between their intersections with opposite sides of the cell contour (Supplementary Fig. 1d). To calculate time-averaged quantities, we normalized trajectories for each generation by the respective division time τ (thus converting each variable to a function of $\phi \equiv t/\tau$). We then split these data into 73 bins, as $\langle\tau\rangle = 73 \pm 7$ min under these conditions, and ensemble-averaged each of these bins over every generation.

We can define five specific values of the width according to the local minima and maxima in the width profile ($w_{\min}$, $w^{st}_{\max}$, $w^{sw}_{\max}$, $w^{st}_{\min}$, $w^{sw}_{\min}$), not all of which may be present in any given cell. Where we identify a primary minimum in the width profile, $w_{\min}$, we can also determine two local maxima, $w^{st}_{\max}$ and $w^{sw}_{\max}$, corresponding respectively to the stalked and swarmer portions (Supplementary Fig. 2a). These values are approximately constant throughout the cell cycle, with $\langle w^{st}_{\max}(\phi)\rangle > \langle w^{sw}_{\max}(\phi)\rangle$. However, at later times ($\phi > 0.6$) the value of $w^{sw}_{\max}(\phi)$ increases until $\langle w^{st}_{\max}(\phi)\rangle \approx \langle w^{sw}_{\max}(\phi)\rangle$. In some cases, additional secondary local minima are observed, w^{st}_{min} and w^{sw}_{min}, corresponding respectively to the stalked and swarmer portions (Supplementary Fig. 2b). Although we note the value of these quantities

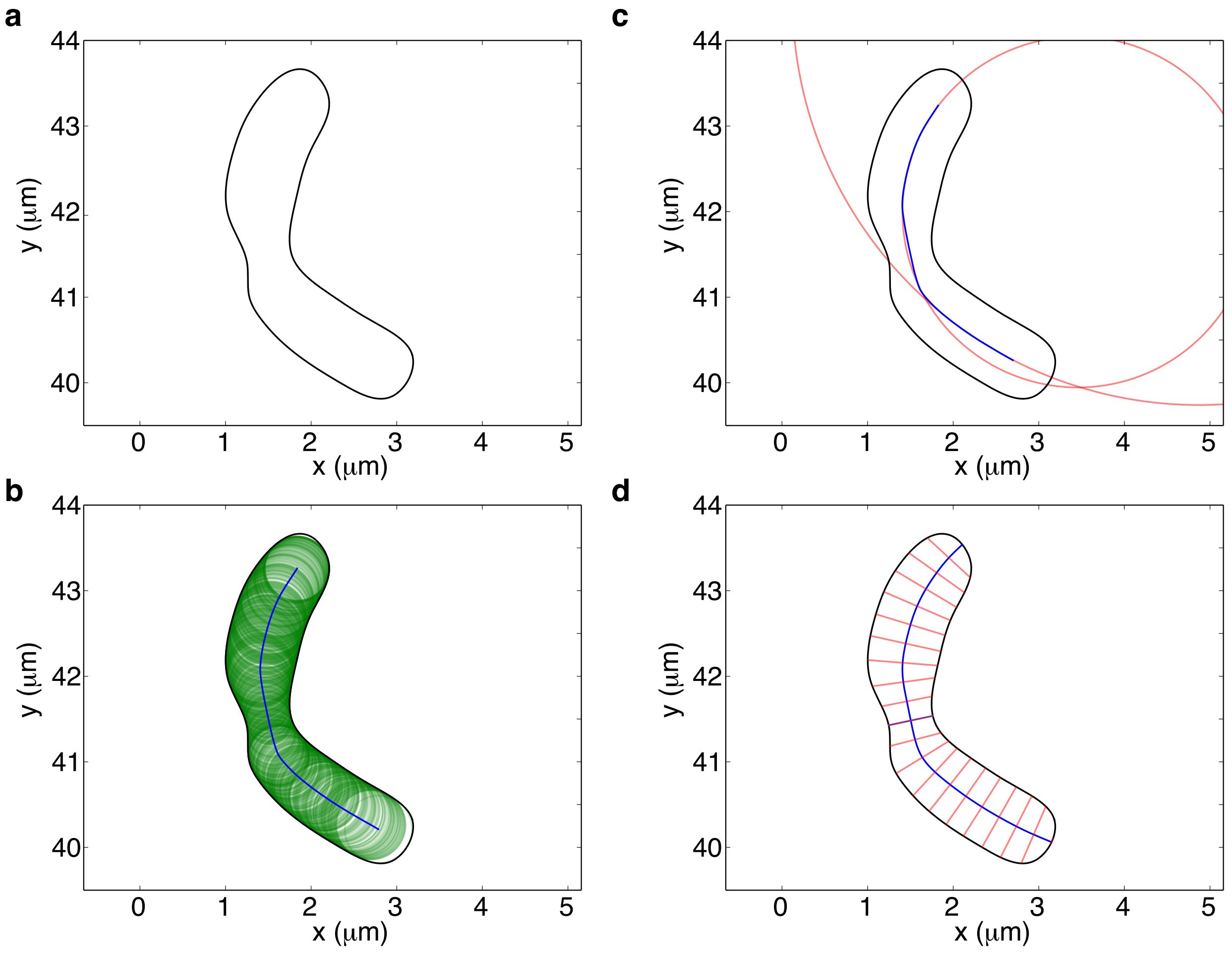

Supplementary Figure 1. **Procedure for calculating shape parameters**. **(a)** B-spline cell contour extracted from segmented phase contrast image. **(b)** The interior part of the medial cell axis was obtained by taking the Voronoi diagram of the cell contour, which is equivalent to the locus of inscribed circles. **(c)** The medial axis located on each side of the width minimum (excluding the constriction zone) was extended to either pole such that average radius of curvature remained constant. **(d)** The cell widths are the ribs perpendicular to the medial axis.

for early times here (where they are approximately equal to their respective local maxima), these minima can only be determined with certainty at later times ($\phi > 0.6$). There, we observe the presence of a secondary minimum in the stalked portions of most cells.

We split each cell into stalked and swarmer portions according to the location of the primary minimum in the width profile. The radius of curvature (R) was calculated as the radius of the best-fit circle to the cell medial axis. We tested two methods of determining the radius of curvature: (I) fitting the whole cell to a circle or (II) fitting the stalked

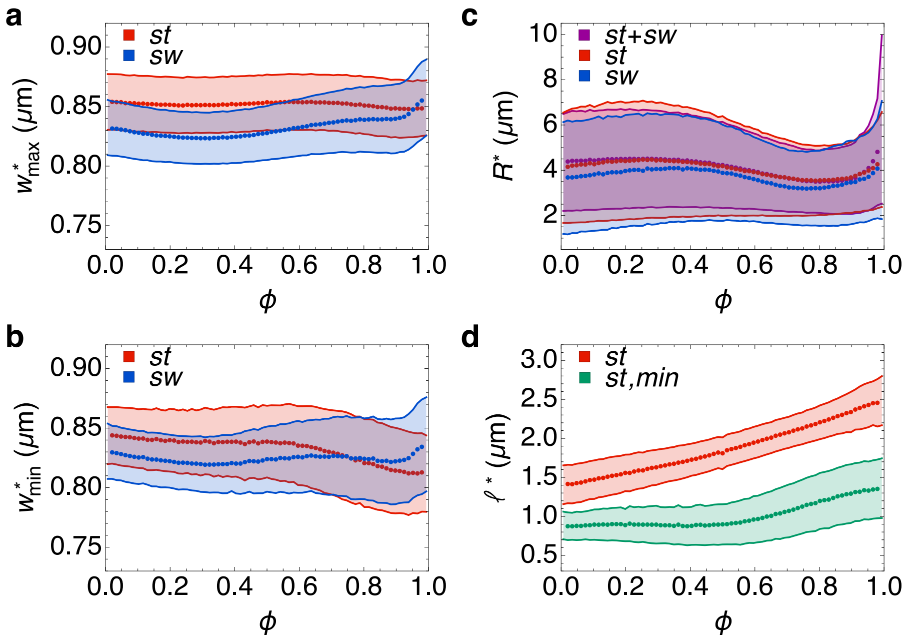

Supplementary Figure 2. **Unnormalized values of the cell shape parameters**. Time-dependence of **(a)** width maxima, and **(b)** width minima, of both stalked and swarmer portions. These data correspond to Fig. 5a in the main text. **(c)** Radius of curvature calculated for either the entire cell (black), the stalked portion only (red), or the swarmer portion only (blue). **(d)** Unnormalized length from stalked pole to either the primary (red) or secondary (green) width minima. These data correspond to Fig. 5b in the main text. Superscript $*$ refers to either st or sw, respectively, as indicated in each figure legend.

and swarmer portions separately. At early times in the cell cycle, (II) gives poorer results because of fewer data points. At later times in the cell cycle, (I) gives poorer results because the stalked and swarmer portions can indeed have differing radii of curvature, which we attribute to the alignment of the swarmer portion of the cell with the direction of fluid flow after the division plane has narrowed enough that it becomes mechanically decoupled from the stalked portion, i.e., like a flexible hinge. However, in the mean the results of (II) are equal to the value calculated by (I) for earlier times. Therefore, we use only data from method (I) but exclude later time points (Supplementary Fig. 2c). Because of the variation in the values of the calculated radius of curvature was large, such that we cannot define a reasonable arithmetic mean without arbitrarily filtering the dataset, we first averaged the corresponding unsigned curvature (equivalent to $\langle R^{-1} \rangle$) and then converted to radius of

	mean ± S.D.		ϕ
$\bar{w}$	0.74 ± 0.02	μm	$[0, 0.5]$
$w^{st}_{\max}$	0.85 ± 0.02	μm	$[0, 0.5]$
$w^{st}_{\min}$	0.84 ± 0.03	μm	$[0, 0.5]$
$w^{sw}_{\max}$	0.83 ± 0.02	μm	$[0, 0.5]$
$w^{sw}_{\min}$	0.82 ± 0.02	μm	$[0, 0.5]$
R	4.44 ± 2.12	μm	$[0, 0.5]$
R^{st}	4.34 ± 2.50	μm	$[0, 0.5]$
R^{sw}	3.94 ± 2.42	μm	$[0, 0.5]$
ℓ^{st}/ℓ	54.3 ± 5.2	%	$[0, 1]$
ℓ^{sw}/ℓ	45.7 ± 5.2	%	$[0, 1]$

Supplementary Table 1. **Average values of the cell shape parameters that are assumed constant over the specified time interval of** ϕ. The time-average value of the width $\langle \bar{w} \rangle$ is smaller than both $\langle w^{st}_{\min} \rangle$ and $\langle w^{sw}_{\min} \rangle$ because it accounts for the width at the primary minimum $w_{\min}$. Note the close correspondence between either width maximum and its corresponding minimum for $\phi < 0.6$, as well as $w^{st}_{\max}$ and $\ell^{st}_{\min}$. Both width and radius of curvature for the swarmer portion are smaller than their respective values for the stalked portion.

curvature (i.e., what we report as $\langle R \rangle$ is actually the harmonic mean, calculated as $\langle R^{-1} \rangle^{-1}$).

The length was also split according to the locations of the local minima, into ℓ^{st} (distance along cell medial axis from stalked pole to $w_{\min}$), ℓ^{sw} (distance along cell medial axis from swarmer pole to $w_{\min}$), $\ell^{st}_{\min}$ (distance along cell medial axis from stalked pole to $w^{st}_{\min}$), and $\ell^{sw}_{\min}$ (distance along cell medial axis from swarmer pole to $w^{sw}_{\min}$). The value of ℓ^{st} and $\ell^{st}_{\min}$ are compared in Supplementary Fig. 2d. Note that the length of the stalked portion ℓ^{st} grows exponentially, with the same time constant as the length ℓ (as does ℓ^{sw}, although it is not shown here for clarity), which is a necessary condition for the addition of peptidoglycan material along the entire length of the cell when the location of $w_{\min}$ is set at early times. At later times ($\phi > 0.6$), the length from stalked pole to secondary minimum $\ell^{st}_{\min}$ also starts to increase.

We relate the asymmetry in the length of stalked and swarmer portions at early times

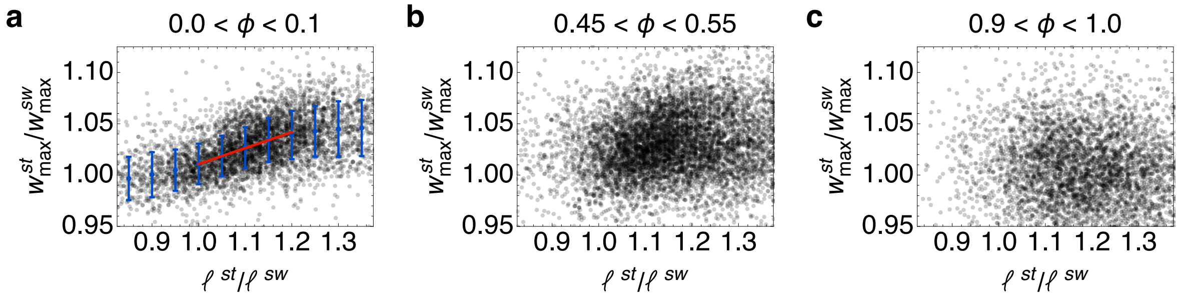

Supplementary Figure 3. **Ratios of maximum width at stalked:swarmer poles versus stalked:swarmer lengths**. The three columns delineate the time period from which data were taken: **(a)** first 10% of the generation, **(b)** middle 10% of the generation, and **(c)** last 10% of the generation. Blue points show the average value of $w^{st}_{\max}/w^{sw}_{\max}$ calculated by binning ℓ^{st}/ℓ^{sw} into bins of width 0.05, with size of each error bar showing the standard deviation of the values within that bin. The best-fit line for $1 \leq \ell^{st}/\ell^{sw} \leq 1.2$ is indicated in red (coefficient of determination $R^2 = 0.15$). This line runs from $(1.0, 1.01)$ to $(1.2, 1.04)$.

to asymmetries in the stalked and swarmer poles. The model predicts a linear relationship between the ratio of lengths ℓ^{st}/ℓ^{sw} and the ratio of the tensions at either pole $\gamma^{st}_p/\gamma^{sw}_p$. We cannot directly measure the latter quantity, but from Laplace's law it is equal to the inverse ratio of the mean curvatures at either pole, which we approximate as the inverse of half the maximum pole width, assuming that the poles are hemispheres with diameter $w^{st}_{\max}$ and $w^{sw}_{\max}$, respectively. Supplementary Fig. 3 shows scatter plots comparing $w^{st}_{\max}/w^{sw}_{\max}$ to ℓ^{st}/ℓ^{sw} for three different time intervals. The red best-fit line is shown only for Supplementary Fig. 3a, for which $R^2 = 0.15$ (linear fits to all other plots produced values of $R^2 < 0.05$). This line runs from the point $(1.0, 1.01)$, corresponding to the dashed blue curve in Fig. 3c of the main text (which is the case of a symmetric cell) to $(1.2, 1.04)$, corresponding to the red solid curve in Fig. 3d of the main text (which is the case of the average asymmetric *C. crescentus* cell). Note that at times $\phi > 0.6$, any correlation disappears.

In order to quantify the error in our width profiles, we imaged a single field of view of 24 *C. crescentus* cells perfused in complex medium at 31°C at a frame rate of 5 frames per second (300 times faster than the frame rate used to acquire all other data), and calculated the splined contours for each cell. We focused in particular on a single "dead" (non-growing and non-dividing) cell, and found the root-mean-square deviation (RMSD) of nearest points along the cell contour between subsequent frames to be 12 nm. In addition, we found the

RMSD of equivalent points along the width profile between subsequent frames to be 28 nm, or a 3.2% pinch depth at the average value of $w^{st}_{\max} = 0.85$ μm.

CELL SHAPE MODEL

Cell Wall Mechanics. The total energy E for the bacterial cell wall is given as the sum of contributions from an active internal pressure P driving cell volume (V) expansion, mechanical energy E_{wall} in the cell wall, and the mechanical energy of interactions with cytoskeletal proteins E_{proteins}:

$$E = -PV + E_{\text{wall}} + E_{\text{proteins}} \ . \tag{S.1}$$

The bacterial cell wall consists of a network of glycan strands cross-linked by peptide chains know as the peptidoglycan network. Growth occurs via the insertion of new peptidoglycan strands into the existing network along with the breaking of existing bonds due to turgor pressure induced stretching. We assume that elastic equilibrium is reached rapidly as compared to the rate of synthesis of new material [2]. As a result of cell wall remodeling and irreversible elongation, growth can be understood as resulting from plastic deformations [3–5]. To understand the origin of the cell wall tension, γ, in the model introduced in the main text, we consider the cell wall as a thin elastic shell that deforms plastically when stretched beyond a maximum strain ε_Y, the yield strain. A thin shell has two modes of elastic deformations, bending and stretching [6], such that $E_{\text{wall}} = E_{\text{stretch}} + E_{\text{bend}}$. In the limit of small thickness of the shell h as compared to its radii of curvature, one can neglect the bending energy (that scales as $E_{\text{bend}} \sim h^3$) whereas the stretching energy E_{stetch} is given by,

$$E_{\text{stretch}} = \frac{h}{2} \int dA \ \sigma_{ij}\varepsilon_{ij} \tag{S.2}$$

where σ_{ij} is the mechanical stress tensor and ε_{ij} is the strain tensor. As yield strain is reached at the onset of growth, we have $\varepsilon_{ij} = \varepsilon_Y \delta_{ij}$ (assuming isotropic stretching), where δ_{ij} is the Kronecker delta. Furthermore, assuming a Hookean constitutive relation for the stress tensor [6]

$$\sigma_{ij} = \frac{Y}{1-\nu^2}\left[(1-\nu)\varepsilon_{ij} + \nu\varepsilon_{kk}\delta_{ij}\right] \ , \tag{S.3}$$

where Y is the Young's modulus and ν is the Poisson ratio, we have $E_{\text{stretch}} = \gamma A$, where,

$$\gamma = \frac{1}{2}\sigma_{ij}\varepsilon_{ij} = \frac{Yh\varepsilon_Y^2}{(1-\nu)} \ . \tag{S.4}$$

For a Gram-negative bacterial cell wall of thickness $h \sim 3$ nm, elastic modulus $Y \sim 40$ MPa [7] and average yield strain $\varepsilon_Y \sim 0.5$, the wall tension is estimated to be $\gamma = 50$ nN/μm. While the actual value for the turgor pressure counteracting this tension can be contested, our choice for the numerical value for internal pressure P can be justified using a simple mechanical argument. Radial force-balance dictates that in order to maintain an average cross-sectional radius $r \simeq 0.4$ μm, a cell wall with surface tension $\gamma \sim 50$ nm/μm has to balance an internal pressure of magnitude $P = 2\gamma/r \simeq 0.25$ MPa, which is numerically very close to our choice for $P = 0.3$ MPa.

Cytoskeletal bundle mechanics. Next, we model the mechanical energy in the cell wall due to interactions with cytoskeletal proteins. Our minimalist approach considers two crucial protein bundles that are directly responsible for maintaining the shape of *C. crescentus* cells. MreB protein bundles contribute an effective energy E_{width}, which favors a rod-like shape, and crescentin filament bundles contribute an energy E_{cres}, which favors a crescent-like shape. We thus have $E_{\text{proteins}} = E_{\text{width}} + E_{\text{cres}}$. MreB subunits form patchy filamentous bundles adherent to the cell wall and oriented perpendicular to the long axis of the cell [8, 9]. The elastic energy stored in an adherent MreB subunit is given by

$$E_{\text{MreB},i} = \frac{k_{\text{b}}}{2} \ell_i \left(\frac{1}{r_i} - \frac{1}{R_m} \right)^2 , \tag{S.5}$$

where i labels the subunit, r_i is the circumferential radius of curvature of the cell wall where the subunit is attached, ℓ_i is the length of the subunit, R_m is the intrinsic radius of curvature and k_b is the bending rigidity of the associated MreB bundle. The total energy imparted by a collection of N_m attached MreB subunits is given by $E_{\text{width}} = \sum_i^{N_m} E_{\text{MreB},i}$. Next, employing a continuum mean field assumption, we replace individual subunit lengths by their average length $\langle \ell \rangle \simeq 5$ nm [10] and assume a uniform number density ρ_m of MreB subunits in the cell surface to obtain

$$E_{\text{width}} = \frac{k_{\text{m}}}{2} \int dA \left(\frac{1}{r} - \frac{1}{R_m} \right)^2 , \tag{S.6}$$

where, $k_m = k_b \rho_m \langle \ell \rangle$ is the effective bending modulus due to MreB induced traction forces. The bending rigidity of an MreB bundle is given by $k_b = k_{\text{MreB}} n^{\xi}$, where k_{MreB} is the flexural rigidity of MreB filaments (assumed to be similar to F-actin), n is the number of MreB protofilaments per bundle, and ξ is an exponent in the range 1–2 depending on the strength of crosslinking or bundling agents [11]. MreB filaments in a bundle appear to have

strong lateral interaction with negligible filament sliding, so we assume $\xi \simeq 2$. The diameter of an MreB protofilament is $\sim$ 4 nm [12] and the average width of an MreB bundle has been determined from super-resolution imaging to be in the range 60-90 nm [13], giving the estimate $n \sim$ 15-22. Estimating the surface number density of MreB subunits as $\rho_m \sim 5\times10^4$ μm^{-2} and using $k_{\text{mreb}} \simeq 10^{-4}$ nNμm^2 [14], we obtain k_m in the range 5.6–12.1 nNμm.

Crescentin proteins form a cohesive bundled structure anchored to the sidewall of *C. crescentus* cells. The energetic contribution due to crescentin is given by

$$E_{\text{cres}} = \frac{k_c}{2}\int ds \left(c(s) - \frac{1}{R_c}\right)^2 \ , \tag{S.7}$$

where k_c is the bending rigidity, s is an arc-length parameter, $c(s)$ is the longitudinal curvature of the cell wall and R_c is the preferred radius of curvature of crescentin bundles. The bending rigidity of crescentin can be expressed as $k_c = Y_c I$, where Y_c is the Young's modulus and I is the area moment of inertia of the bundle (with width w_c) given by $I = \pi w_c^4/64$. Since crescentin is an intermediate filament homologue, we assume that Y_c is similar to the Young's modulus of intermediate filament bundles given by $Y_c \sim 300$ MPa [15]. Assuming $w_c \sim 0.1$ μm, we estimate the bending rigidity of a crescentin bundle to be $k_c \sim 1.5$ nNμm^2.

Mean field model for cell shape and size dynamics. As described in the main text, the total mechanical energy in the cell wall of *C. crescentus* cells can be given as a sum of contributions from internal pressure, wall surface tension, mechanical energy of interactions with bundles of cytoskeletal proteins such as crescentin and MreB, and the constriction energy E_{div} during cell division. In the mean field description, we neglect the contributions from E_{div} and disregard any spatial variations in cell geometry. The mean field description is a good approximation of the cell shape dynamics for $\phi < 0.5$, where the average width and the midline radius of curvature remains constant (Fig. 2 in the main text). We approximate the shape of *C. crescentus* cells as the segment of a torus with radius of curvature $R(t)$, cross-sectional width $\bar{w}(t)$ and spanning angle $\theta(t)$. We also neglect the pole caps that are mechanically rigid and do not remodel during wall growth. The total energy is then given by

$$E[\bar{w}, R, \theta] = -P(\pi\bar{w}^2 R\theta)/4 + \gamma(\pi R\bar{w}\theta) + E_{\text{width}} + E_{\text{cres}} \ , \tag{S.8}$$

where

$$E_{\text{width}} = \frac{k_m}{2}(\pi R w\theta)\left[(\bar{w}/2)^{-1} - R_m^{-1}\right]^2 \ , \tag{S.9}$$

$$E_{\rm cres} = \frac{k_c}{2}(R - \bar{w}/2)\theta \left[(R - \bar{w}/2)^{-1} - R_c^{-1}\right]^2 \ . \tag{S.10}$$

From the above expressions, we see that the total energy has the scaling form $E[\bar{w}, R, \theta] = \theta U[\bar{w}, R]$. According to Eq. (1) in the main text, the dynamics of the radius of curvature R, the spanning angle θ and the width w are given by

$$\frac{1}{R}\frac{dR}{dt} = -\Phi_R \frac{\partial E}{\partial R} \ , \tag{S.11a}$$

$$\frac{1}{\bar{w}}\frac{d\bar{w}}{dt} = -\Phi_w \frac{\partial E}{\partial \bar{w}} \ , \tag{S.11b}$$

$$\frac{1}{\theta}\frac{d\theta}{dt} = -\Phi_\theta \frac{\partial E}{\partial \theta} \ , \tag{S.11c}$$

where Φ_R, Φ_θ and Φ_w are the rate constants. The steady-state values for the radius of curvature of the centerline and the cross-sectional width is given by the solutions to $\partial_R U = 0$ and $\partial_w U = 0$. As shown in Supplementary Fig. 4a the energy density U admits a stable absolute minimum in width, controlled by the stiffness parameter k_m such that for lower values of k_m (red curve in Supplementary Fig. 4a), U does not have a minimum and the width grows in time rendering the rod-like shape unstable. Similarly, the parameter k_c controls cell curvature, such that the energy density has a stable absolute minimum in R beyond a critical stiffness k_c (Supplementary Fig. 4b). At lower values of k_c the cell does not have a stable radius of curvature and the energy is minimized by increasing R. This leads to straightening of the cell's medial axis. In the parameter range where the cell maintains a stable value for $\bar{w}$ and R, the energy density U becomes a constant during cell elongation. From the growth law introduced in the main text, the spanning angle θ evolves in time according to the equation

$$\frac{d\theta}{dt} = -(\Phi_\theta U)\theta(t) \ , \tag{S.12}$$

such that the condition for exponential growth becomes $U < 0$.

Constriction dynamics. As described in the main text, we assume that the shape of the constriction zone is given by two intersecting hemishperical segments with diameter $w_{\rm max}$. The total surface area of the septum is given by $S(t) = \pi w_{\rm max} \ell_s(t)$, where $\ell_s(t)$ is the total length of the spherical segments (see Supplementary Fig. 5a). Using elementary geometry, we obtain the following relation between $\ell_s(t)$ and the minimum width, $w_{\rm min}(t)$:

$$w_{\rm min}(t) = w_{\rm max}\sqrt{1 - (\ell_s(t)/w_{\rm max})^2} \ . \tag{S.13}$$

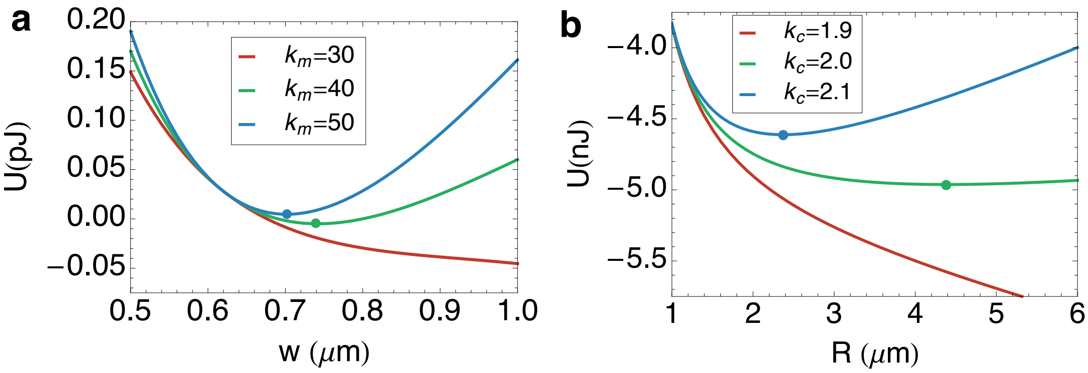

Supplementary Figure 4. **Dependence of the mechanical energy on cell width and radius of curvature**. **(a)** Energy density U as a function of mean cell width $\bar{w}$ at various values of bending stiffness (in units of $nN\mu$m): $k_m = 30$ (red), $k_m = 40$ (green) and $k_m = 50$ (blue), for a fixed radius of curvature $R = 4.4$ μm and $k_c = 2$ nNμm^2. The absolute minima (if it exists) are indicated by solid circles. **(b)** U as a function of radius of curvature R at various values of crescentin stiffness (in units of $nN\mu$m^2): $k_c = 1.9$ (red), $k_c = 2.0$ (green) and $k_c = 2.1$ (blue), for a fixed width $\bar{w} = 0.74$ μm and $k_m = 40$ nNμm. Other parameters: $P = 0.3$ MPa, $\gamma = 50$ nN/μm, $R_c = 0.5$ μm, $R_m = 0.31$ μm.

If septal growth occurs by addition of new peptidoglycan strands while maintaining the curvature of the spherical segments, then $\ell_s(t)$ is the shape variable controlling the growth of septal surface area S as well as the constriction dynamics of $w_{\min}(t)$. To describe the dynamics of $\ell_s(t)$, we assume that initial phase of elongation occurs with a velocity v_0, and thereafter growth follows an exponential law with rate κ_d,

$$\frac{d\ell_s}{dt} = v_0 + \kappa_d \ell_s(t) \; . \qquad \text{(S.14)}$$

Multiplying both sides of the equation by $\pi w_{\max}$, we derive the growth dynamics of the surface area S(t),

$$\frac{dS}{dt} = \kappa_0 + \kappa_d S(t) \; , \qquad \text{(S.15)}$$

where $\kappa_0 = \pi w_{\max} v_0$. Having determined the dynamics of $w_{\min}$, one can evaluate the time-dependence of the average width $\bar{w}$ defined as, $\bar{w}(t) = \frac{1}{\ell(t)} \int_0^{\ell(t)} w(u,t) du$, where u is the coordinate along the centerline. Using the simplified geometry of the constricting cell, given by two toroidal segments (peripheral regions) connected by two intersecting hemispheres

(septal region), one gets,

$$\bar{w}(t) = \frac{1}{l(t)}\left[\bar{w}(\ell(t) - \ell_s(t)) + 2\int_0^{\ell_s(t)/2} w_s(u,t)du\right] , \tag{S.16}$$

where $w_s(u) = w_{\max}\sqrt{1-(2u/w_{\max})^2}$, is the width in the septal region and $\bar{w}$ is the average width of the stalked and swarmer components as determined by Eq. (S.11b). As shown in Fig. 2c in the main text (blue solid line), Eq. (S.16) is in excellent agreement with the experimental data and captures the dip in $\bar{w}(t)$ seen for $\phi > 0.5$. Fig. 2b in the main text also shows that the radius of curvature of the centerline ($R(t)$) drops for $\phi > 0.5$ when cell constriction is prominent. Therefore we examine the role of constriction dynamics on the time-dependence of the centerline radius of curvature.

The centerline spans a distance $\ell_s(t)$ and an angle $\theta_s(t)$ in the septal region, as shown in Supplementary Fig. 5a. Consider a small segment $\delta\ell_s(t)$ along the centerline with a local radius of curvature R and spanning angle $\delta\theta_s$. We then have the relation $\delta\ell_s(t) = R(t)\delta\theta_s(t)$. This leads to the following identity,

$$\frac{1}{\delta l_s}\frac{d(\delta l_s)}{dt} = \frac{1}{R}\frac{dR}{dt} + \frac{1}{\delta\theta_s}\frac{d(\delta\theta_s)}{dt} . \tag{S.17}$$

Now the geometry of the septal region (Supplementary Fig. 5a) directly relates the rate of increase in the spanning angle $\delta\theta_s(t)$ to the rate of drop in the tangent angle ($\psi(t)$) at the constriction site, $\frac{1}{\delta\theta_s}\frac{d(\delta\theta_s)}{dt} = -\frac{1}{\delta\psi}\frac{d(\delta\psi)}{dt}$. Furthermore, using the relations, $w_{\max}\sin\psi(t) = w_{\min}(t)$ and $w_{\max}\cos\psi(t) = l_s(t)$, we get $\delta\psi(t) = \delta w_{\min}(t)/l_s(t) = -\delta l_s(t)/w_{\min}(t)$. The last equality follows after variations of Eq. (S.13). As a result we have the following kinetic relation,

$$\frac{d(\delta\psi)/dt}{\delta\psi} = \frac{dw_{\min}/dt}{w_{\min}} - \frac{d(\delta l_s)}{\delta l_s} = -\frac{d(\delta\theta_s)/dt}{\delta\theta_s} . \tag{S.18}$$

Combining the identities in Eq. (S.17) and (S.18) we get, $R^{-1}dR/dt = w_{\min}^{-1}dw_{\min}/dt$, showing that the radius of curvature drops at the same rate as $w_{\min}(t)$ shrinks.

Contour Model for cell shape. To quantitatively capture the experimentally observed spatial variations in cell shape we study an effective two-dimensional contour model for the cell shape. This approach facilitates closer comparison with the two-dimensional splined contours obtained from our experimental data. The contour description of the rod-like cell shape negelects circumferential variations in cell geometry. The model incorporates non-uniform materials properties and mechanical constraints across the cell wall, with the poles

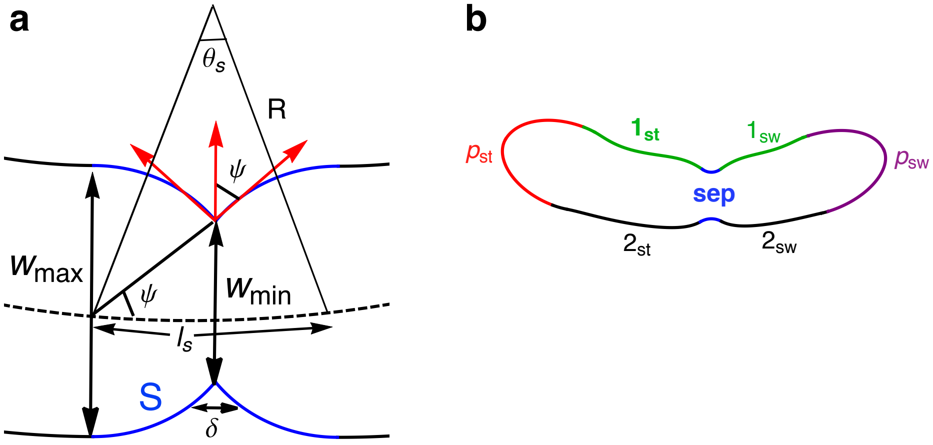

Supplementary Figure 5. Geometry of a constricting cell contour. (a) Schematic of the constriction zone of a dividing cell where the dashed line indicates the centerline with length l_s, radius of curvature R and spanning angle θ_s. S (blue) denotes the septal cell wall, ψ is the tangent angle at the constriction site and δ is the width of the division ring. **(b)** Compartmentalizing the cell contour into the (1) stalked pole p_{st} (red), (2) swarmer pole p_{sw} (purple) (3) upper wall $1_{st/sw}$ (with crescentin bundle attached, green), (4) Lower wall $2_{st/sw}$ (black) and the (5) Septal region (blue).

and the septal region being mechanically stiffer than the rest of the cell wall. By exploiting the rod-like geometry, one can use the centerline curve to divide the contour into two parts, the *upper* and the *lower* cell wall. As shown in Supplementary Fig. 5b we further subdivide the cell contour into the stalked and swarmer poles ($p_{st/sw}$), the upper ($1_{st/sw}$) and lower cell wall ($2_{st/sw}$), and the septal region (sep).

We parametrize the instantaneous shape of the cell contour using the two-dimensional centerline curve $\mathbf{R}(u,t)$, the length of the centerline $l(t)$ and the width $w(u,t)$, where u is the absolute distance along the centerline from the stalked pole such that $u \in [0, l(t)]$. If $\hat{\mathbf{n}}$ denotes the outward unit normal vector on the centerline, the curves defining the upper and lower parts of the cell contour, $\mathbf{r}_\pm(u,t)$, are given by the relation, $\mathbf{r}_\pm = \mathbf{R} \pm w_\pm \hat{\mathbf{n}}$, where $w_\pm(u)$ represent the perpendicular distances of the top and bottom curves from the centerline. The total cell width is then given by $w(u,t) = w_+(u,t) + w_-(u,t)$. It is convenient to switch to polar coordinates, where the shape of the cell contour is given by the re-parametrized curve $\mathbf{r}_\pm(\varphi,t)$, where $0 < \varphi < \theta(t)$ is the angular coordinate spanning the centerline, which can be approximated as the arc of a circle with radius $R(t)$. Since the ratio $w_\pm/R$ ($\simeq 0.1$) is

small at all times, one can approximate the local curvature as

$$c_{\pm}(\varphi,t) = \frac{1}{R(t)}\left(1 - \frac{w'_{\pm}(\varphi,t) + w''_{\pm}(\varphi,t)}{R(t)}\right) + \mathscr{O}(w_{\pm}^2, w_{\pm}'^2, w_{\pm}''^2)\ , \tag{S.19}$$

where prime denotes derivative with respect to φ and the subscripts $\pm$ represent the upper and the lower part of the cell contour respectively. Furthermore, in the linear regime, the differential arc length can be approximated as $du \simeq R d\varphi$. The dynamics of the shape parameters $l(t)$, $\theta(t)$, and $R(t)$ are determined from the kinetic law in Eq. (1) of the main text. The instantaneous width profile $w(u,t)$ results from minimizing the total energy functional, which leads to the following shape equation,

$$P - \gamma(u)c_{\pm}(u,t) + f_{\text{width}}(u,t) + f_{\text{cres}}(u,t) = 0\ , \tag{S.20}$$

where, $\gamma(u)$ is the tension on the cell contour and $f_{\text{width}}(u,t)$ and $f_{\text{cres}}(u,t)$ are the linear force densities on the contour due to maintenance of width and the crescent shape, respectively. In the contour model we simplify the energetic contribution due to maintenance of width (acting on the sections $1_{st/sw}$ and $2_{st/sw}$) as

$$E_{\text{width}} = \frac{K_m}{2}\int du\,(w_{\pm} - R_m)^2\ , \tag{S.21}$$

where the elastic constant K_m depends on the bending rigidity k_m introduced in the main text as $K_m = k_m/R_m^4$. This linear approximation holds if $(w_{\pm} - R_m)/R_m \ll 1$. From our data and mean field model fits we get $(w_{\pm} - R_m)/R_m \simeq 0.15$. The resultant force density is given by $f_{\text{width}}(u,t) = -K_m\,(w_{\pm}(u,t) - R_m)$.

The bending energy induced by crescentin protein bundles anchored onto the cell wall (regions $1_{st/sw}$) is given by

$$E_{\text{cres}} = \frac{k_c}{2}\int ds\,[c_{+}(u) - c_0]^2\ , \tag{S.22}$$

where $c_0 = 1/R_c$ is the spontaneous curvature of the crescentin bundle, and s is the arc-length parameter along the upper part of the cell contour ($1_{st/sw}$) which is related to u as $ds = du - w_{+}d\varphi$. To obtain the force density we consider an infinitesimal deformation, δr, of the upper contour as $\mathbf{r}_{+} \to \mathbf{r}_{+} + \mathbf{n}_{+}\delta r$, where $\mathbf{n}_{+}$ is the outward unit normal. Accordingly the curvature and the differential arc-length changes, $c_{+} \to c_{+} + \delta c_{+}$ and $ds \to ds + \delta(ds)$, where $\delta c_{+} = c_{+}^2\delta r + d^2(\delta r)/ds^2$ and $\delta(ds) = -c_{+}ds$ [16]. The resultant force density is obtained after

variations of the energy functional, $\delta E_{\rm cres} = \int f_{\rm cres}\delta r ds$, where $f_{\rm cres}\delta r ds = \delta[(c_+ - c_0)^2 ds]$. This leads to the following non-linear force contribution:

$$f_{\rm cres} = \frac{k_c}{2}\left(2\frac{d^2c_+}{ds^2} + c_+^3 - c_+c_0^2\right) \ . \tag{S.23}$$

Using Eq. (S.19), $f_{\rm cres}$ can be linearized and expressed using the angular coordinate φ as

$$f_{\rm cres}(u,t) = \frac{k_c}{R^4}\left[-w_+''''(u,t) + \frac{1}{2}(c_0^2R^2 - 5)w_+''(u,t) + \frac{1}{2}(c_0^2R^2 - 3)w_+(u,t) + R(1 - c_0^2R^2)\right] \ . \tag{S.24}$$

Compartmentalizing the cell contour. (1) Pole caps: The cell poles are assumed to be mechanically inert in the sense that they do not interact with the active cytoskeletal proteins such that $f_{\rm width} = f_{\rm cres} = 0$. The mechanical forces acting on the cell poles come from turgor pressure P, and the tension γ_p. The shape of the cell poles are then described by the two-dimensional Laplace's law,

$$\gamma_p^{st,sw}c_\pm(u,t) = P \ , \tag{S.25}$$

where the superscripts st and sw denote respectively the stalked and swarmer poles.

(2) Upper cell contour ($1_{st/sw}$): The region $1_{st/sw}$ in the upper cell contour obeys the force-balance equation:

$$P - \gamma c_+(u,t) + f_{\rm width}(u,t) + f_{\rm cres}(u,t) = 0 \ . \tag{S.26}$$

At the endpoints of the segments $1_{st/sw}$ we impose the boundary condition that the curvature c must equal the longitudinal curvature of the poles.

(3) Bottom cell contour ($2_{st/sw}$): In the absence of crescentin, the region $2_{st/sw}$ in the bottom cell contour obeys the force-balance equation:

$$P - \gamma c_-(u,t) + f_{\rm width}(u,t) = 0 \ . \tag{S.27}$$

(4) Septal region: We incorporate the effect of constriction in the cell shape equation by imposing the constraint (boundary condition) that $w(\ell^{st}(t),t) = w_{\rm min}(t)$, where $w_{\rm min}(t)$ evolves according to the kinetics described in Eq. (6) and (7) of the main text. Furthermore, the curvatures at the end points of the septal segments must conform to the curvature of the newly formed poles. Subject to these boundary conditions, the width profile in the upper septal region ($\ell^{st} - \ell_s/2 < u < \ell^{st} + \ell_s/2$) is given by,

$$P - \gamma_s c_+(u,t) + f_{\rm width}(u,t) + f_{\rm cres}(u,t) = 0 \ , \tag{S.28}$$

with a surface tension (γ_s) of the newly formed poles chosen to be much higher than the peripheral region, $\gamma_s \simeq 4.5\gamma$. The contour below the centerline obeys the equation,

$$P - \gamma_s c_-(u,t) + f_{\text{width}}(u,t) = 0 \ . \tag{S.29}$$

The governing cell shape equation given in Eq. (S.20), is then solved numerically in each part of the cell contour with matching boundary conditions in $w_\pm(u,t)$ and its derivatives.

SUPPLEMENTARY REFERENCES

---

[1] Brandt, J. W. Convergence and continuity criteria for discrete approximations of the continuous planar skeleton. *CVGIP-Imag Understan* **59**, 116–124 (1994).

[2] Goriely, A., Robertson-Tessi, M., Tabor, M. & Vandiver, R. Elastic growth models. In Mondaini, R. P. & Pardalos, P. (eds.) *Mathematical Modelling of Biosystems*, 1–44 (Springer-Verlag, Berlin, 2008).

[3] Cosgrove, D. J. Cell wall yield properties of growing tissue evaluation by in vivo stress relaxation. *Plant Physiol* **78**, 347–356 (1985).

[4] Koch, A. L. *Bacterial Growth and Form* (Springer Science+Business Media, Dordrecht, 2001).

[5] Boudaoud, A. Growth of walled cells: from shells to vesicles. *Phys Rev Lett* **91**, 018104 (2003).

[6] Landau, L. D. & Lifshitz, E. *Course of Theoretical Physics Vol 7: Theory and Elasticity* (Pergamon Press, London, 1959).

[7] Boulbitch, A., Quinn, B. & Pink, D. Elasticity of the rod-shaped gram-negative eubacteria. *Phys Rev Lett* **85**, 5246 (2000).

[8] Garner, E. C. *et al.* Coupled, circumferential motions of the cell wall synthesis machinery and MreB filaments in *B. subtilis*. *Science* **333**, 222–225 (2011).

[9] Gahlmann, A. & Moerner, W. Exploring bacterial cell biology with single-molecule tracking and super-resolution imaging. *Nat Rev Microbiol* **12**, 9–22 (2014).

[10] Colavin, A., Hsin, J. & Huang, K. C. Effects of polymerization and nucleotide identity on the conformational dynamics of the bacterial actin homolog MreB. *Proc Natl Acad Sci USA* **111**, 3585–3590 (2014).

[11] Bathe, M., Heussinger, C., Claessens, M. M., Bausch, A. R. & Frey, E. Cytoskeletal bundle mechanics. *Biophys J* **94**, 2955–2964 (2008).

[12] van den Ent, F., Amos, L. A. & Löwe, J. Prokaryotic origin of the actin cytoskeleton. *Nature* **413**, 39–44 (2001).

[13] Reimold, C., Soufo, H. J. D., Dempwolff, F. & Graumann, P. L. Motion of variable-length mreb filaments at the bacterial cell membrane influences cell morphology. *Mol Biol Cell* **24**, 2340–2349 (2013).

[14] Gittes, F., Mickey, B., Nettleton, J. & Howard, J. Flexural rigidity of microtubules and actin filaments measured from thermal fluctuations in shape. *J Cell Biol* **120**, 923–934 (1993).

[15] Guzman, C. *et al.* Exploring the mechanical properties of single vimentin intermediate filaments by atomic force microscopy. *J Mol Biol* **360**, 623–630 (2006).

[16] Mumford, D. Elastica and computer vision. In Bajaj, C. L. (ed.) *Algebraic Geometry and its Applications*, 491–506 (Springer-Verlag, New York, 1994).
